# Cost awareness among intensivists in their daily clinical practice: a prospective multicentre study

**DOI:** 10.1007/s10198-024-01686-y

**Published:** 2024-03-13

**Authors:** Timothée Lehut, Céline Lambert, Romain Mortier, Emmanuel Futier, Russell Chabanne, Ulrich Bauer, Philippe Verdier, Ramin Ravan, Philippe Ocquidant, Charline Mourgues, Alexandre Lautrette

**Affiliations:** 1 Department of Anaesthesiology and Critical Care Medicine, CHU Clermont-Ferrand, Clermont-Ferrand, France; 2 Biostatistics Unit, DRCI, CHU Clermont-Ferrand, Clermont-Ferrand, France; 3 Intensive Care Unit, Cancer Center Jean Perrin, Clermont-Ferrand, France; 4 Intensive Care Unit, Centre Hospitalier de Montluçon, Montluçon, France; 5 Intensive Care Unit, Centre Hospitalier Jacques Lacarin, Vichy, France; 6 Intensive Care Unit, CHU de la Réunion, La Réunion, France; 7 Intensive Care Medicine, CHU Clermont-Ferrand,Intensive Care Unit, Cancer Centre Jean Perrin, Clermont-Ferrand, 54 rue Montalembert BP69, Cedex 1, 63003 France; 8 LMGE (Laboratoire Micro-organismes: Génome et Environnement), UMR CNRS 6023, Université Clermont Auvergne, Clermont-Ferrand, France

**Keywords:** Cost awareness, Medical knowledge, Daily clinical practice, Sustainable hospital development, Financial impact, Health care cost control

## Abstract

**Background:**

Better cost-awareness is a prerogative in achieving the best benefit/risk/cost ratio in the care. We aimed to assess the cost-awareness of intensivists in their daily clinical practice and to identify factors associated with accurate estimate of cost (50–150% of the real cost).

**Methods:**

We performed a prospective observational study in seven French ICUs. We compared the estimate of intensivists of the daily costs of caring with the real costs on a given day. The estimates covered five categories (drugs, laboratory tests, imaging modalities, medical devices, and waste) whose sum represented the overall cost.

**Results:**

Of the 234 estimates made by 65 intensivists, 70 (29.9%) were accurate. The median overall cost estimate (€330 [170; 620]) was significantly higher than the real cost (€178 [124; 239], *p* < 0.001). This overestimation was found in four categories, in particular for waste (€40 [15; 100] vs. €1.1 [0.6; 2.3], *p* < 0.001). Only the laboratory tests were underestimated (€65 [30; 120] vs. €106 [79; 138], *p* < 0.001). Being aware of the financial impact of prescriptions was factor associated with accurate estimate (OR: 5.05, 95%CI:1.47–17.4, *p* = 0.01). However, feeling able to accurately perform estimation was factor negatively associated with accurate estimate (OR: 0.11, 95%CI: 0.02–0.71, *p* = 0.02).

**Conclusion:**

French intensivists have a poor awareness of costs in their daily clinical practice. Raising awareness of the financial impact of prescriptions, and of the cost of laboratory tests and waste are the main areas for improvement that could help achieve the objective of the best care at the best cost.

**Supplementary Information:**

The online version contains supplementary material available at 10.1007/s10198-024-01686-y.

## Background

In France, the annual consumption of medical care amounted to 226.7 billion euros in 2021, equivalent to 9.1% of gross domestic product [1], one of the highest shares of gross domestic product devoted to health spending worldwide [2]. Few countries reach this rate. Some emerging economies are achieving this because of financing reforms to their health system to achieve universal health coverage [3]. The French health system is not in a context of substantial reforms. Although this high gross domestic product is laudable, it represents a heavy burden on the country. The current objective is to maintain a high-quality healthcare offering for the French population while controlling expenses. Clinicians are directly involved in health expenditure since they decide on the prescription of examinations and drugs, which are more or less expensive, effective or appropriate and sometimes result in avoidable adverse events [4]. Knowing the beneficial or adverse effect of an examination or drug but also the cost can influence its prescription. Good medical practice requires the use of diagnostic or therapeutic resources with the best benefit/risk/cost ratio. The intensive care unit (ICU) is a very costly department whose running can sometimes reach up to 20% of the overall hospital budget [5]. Studies of intensivists’ knowledge of costs are scarce [6–10]. However, they all reported that intensivists had a poor knowledge of the theoretical costs of the main drugs and examinations commonly prescribed in the ICU. A drug prescription can also include single-use medical devices, which generates waste. Another form of waste can result from the prescription of unnecessary or repeated examinations. Avoiding waste in intensive care fits perfectly into the “less is more” approach which, in accordance with the current guidelines recommends not multiplying prescriptions, dosages and invasive interventions so as not to harm the patient [11–13]. This attitude of “reasonable or sober medicine” underlines the increasingly strong desire for sustainable hospital development [14].

To our knowledge, there has been no assessment of cost-awareness in intensivists in their daily clinical practice, which would be the first step in identifying areas to educate them on the importance of a culture “of the best care at the best cost”. The aim of this study was to assess the cost-awareness of intensivists in their daily clinical practice and to identify factors associated with an accurate estimation of cost.

## Methods

### Study design and data patients

We conducted a prospective multicentre observational study from March 1 to October 31, 2022 in seven ICUs in France, the medical-surgical, neurosurgical, and surgical ICUs at the teaching hospital of Clermont-Ferrand, and the ICUs of Vichy hospital, Montluçon hospital, and the Clermont-Ferrand Cancer centre, and in the neurosurgical ICU of the Saint-Pierre teaching hospital on Reunion Island. The study was approved by the “Est IV” personal protection committee. (No. 2021/CE61; no. IRB: 00008526) and declared to the National Commission for Computing and Freedoms (No. M200603). It followed the STROBE (STrengthening the Reporting of Observational studies in Epidemiology) recommendations for good clinical research practice (Additional file 1: Appendix 1).

The participants were junior and senior intensivists who performed clinical activities in the ICU on the day of data collection. The exclusion criteria were refusal to participate in the study and prior participation in the study. The collection day was a weekday chosen randomly from 8 a.m. to 6 p.m. All data were collected by a single investigator (TL). Information on the objective of the study and on the data collected was given to the paramedical staff just before the start of the data collection day. Information on the objective of the study and on the data collected was only communicated to the medical team at the end of the data collection day in order not to change their prescription habits and to avoid a search for information on the cost of care which could have distorted the spontaneous assessment of the participants.

### Data collection

Two sets of data were collected for this study, the real costs of the care of ICU patients and the costs estimated by the intensivists. For the first set, details of the various drugs prescribed and the laboratory tests and imaging modalities performed were collected from the computer software used for all prescriptions. Data on medical devices were collected directly by the paramedical staff from record books in patient rooms. The investigator randomly checked the correct completion of these files throughout the collection day. Waste data were collected directly by the investigator.

Five cost categories were analysed. Drug costs were based on all treatments (per os, intravenous, subcutaneous, enteral and parenteral nutrition, and transfusion) administered to the patient from 8 a.m. to 6 p.m. For continuous infusion treatments, the dose of drug used was calculated according to the speed of the syringe pump (in ml/h), the number of hours of administration, and adjusted in the event of a change in speed. Oxygen consumption was not collected.

The costs of the laboratory tests were principally for tests carried out between 8 a.m. and 6 p.m., including those sent to the laboratory and those performed by an ICU biological analyser. The very first tests of the day, performed between 6 a.m. and 8 a.m. depending on the habits of each ICU, were also taken into account in the collected data since they were used by the medical team during the working day. This information was explained to intensivists when they came to estimate the costs.

The costs of imaging modalities were based on all imaging including radiology, ultrasound, computed tomography-scan and magnetic resonance imaging performed for patients on the collection day. Ultrasounds were only taken into account in the collected data when the examination was performed by a radiologist. Again, this information was explained to intensivists when they estimated the costs.

Medical device costs were based on all single-use materials that were used for administration of treatment including sterile and non-sterile compresses, sterile drapes, syringes, needles, peripheral and central venous catheters, arterial catheters, tracheostomy kits, dressings, mouth care kits, mouth guards, bladder catheterization kits, penis sheaths, ring fittings, tracheal tubes, attachment straps, cannulas, stylets, oxygen masks and goggles, nebulizers, tubing ventilation, sampling lines, sterile collectors, Combicath® kits, filters, sterile water bottles, betadine bottles, dressings, probe covers, sterile ultrasound gel bags, disinfection kits for endoscopes, intracranial pressure sensors and fitting kits, sterile gowns, sterile and non-sterile gloves, scalpels, bladder catheters, nasogastric catheters, laryngoscope blades, rectal catheters, tubing, extension lines, stopcocks, stoppers, non-return valves, and transfusion lines. Each single-use material was collected with its brand, type and size to obtain its exact price. Reusable material was not taken into account. Boxes of numerous individual medical devices, such as packs of 100 non-sterile gloves, were counted when they were renewed.

Waste costs were based on a measurement of the total weight of waste generated for each patient on the day of collection. The bins present in each patient’s room were weighed by the investigator at 8 a.m. and at 6 p.m. and each time they were changed during the day. Waste equivalent to household waste and waste from healthcare activities with infectious risks were weighed separately since their management cost is not the same.

The real costs of drugs and medical devices were retrieved from the price lists from the supply pharmacy. The costs of the different types of waste differed between hospitals. The cost of laboratory tests was based on the 59th version of the nomenclature of laboratory test procedures of the French national health insurance scheme. The cost of imaging modalities was based on the 70th version of the common classification of medical procedures in radiology.

The intensivists accepting to make cost estimations received information on the objective of the study and on the data collected at the end of the collection day. They then answered an anonymous questionnaire composed of two parts, of which the first included demographic data on age, professional status (junior or senior), number of years of ICU experience, and courses, seminars or any other training related to cost estimation. They then carried out a self-assessment using visual analogue scales ranging from 0 (entirely incapable/insensitive) to 100 (very capable/sensitive) on their ability to estimate the costs of their prescription, their awareness of the financial impact of their prescriptions (*ceteris paribus*) and their awareness of the environmental impact of their prescriptions (*ceteris paribus*). The self-assessments were divided into three score categories: <40/100 corresponding to low ability or awareness; 40–60/100 corresponding to intermediate ability or awareness; >60/100 corresponding to high ability or awareness. The second part of the questionnaire included the cost estimates. Participants only estimated the costs of the patients they had cared for throughout the day. The estimates focused on the cost related to the five categories mentioned above: drugs, laboratory tests, imaging modalities, medical devices and waste. The participants had the possibility of consulting the software of their prescriptions (drugs, examinations and others) but were not allowed to check the cost of each of the items or to communicate with one another. The costs were estimated in euros.

### Statistical analysis

The sample size was estimated according to Cohen’s recommendations, which define effect size (ES) bounds as small (ES: 0.2), medium (ES: 0.5) and large (ES: 0.8). In order to highlight a significant effect size of 0.8 between the overall real cost and the overall estimated cost, 19 doctors were necessary, with a two-sided type I error of 5% and a 90% power (G*Power, difference of two paired means).

Statistical analyses were performed with Stata software (version 15; StataCorp, College Station, Texas, USA). All tests were two-sided, with an alpha level set at 5%. Categorical variables were expressed as counts and associated percentages, and continuous variables as mean ± standard deviation or median [25th ; 75th percentiles], with regard to their statistical distribution. The real and estimated costs (overall and by category) were compared by linear mixed models after logarithmic transformation with the centre and the intensivist as random effects. Results were expressed as Hedge’s ES and 95% confidence interval (CI). The factors associated with the difference between the overall real cost and the overall estimated cost (age, sex, experience, senior/junior status, etc.) were also studied by linear mixed models. Then, the estimates were categorized according to whether they were accurate (between 50 and 150% of the real cost) or not. The factors associated with an accurate estimation (overall and by category) were studied by mixed effects logistic regressions, with the centre and the intensivist as random effects. The results were presented as odds ratio (OR) and 95% CI.

## Results

No intensivist refused to participate in the study. A total of 65 intensivists, 34 junior doctors and 31 senior, completed the questionnaire. Their average age was 33.6 ± 9.6 years. Only two senior intensivists had received training. The characteristics of the intensivists are shown in Table 1. Thirty-four intensivists assessed three patients, 23 intensivists four patients, and 8 intensivists five patients, giving a total of 234 analysed estimates obtained from 133 different patients. The median of the real overall cost per patient was €174 [120; 237]. The cost of each category for the 133 patients is given in Table 2.

**Table 1:** Characteristics of the intensivists

	(*n* = 65)
Intensive care units	
Cancer centre (Clermont-Ferrand)	12 (18.5)
Neurosurgical ICU (Reunion Island)	13 (20.0)
Medical-surgical ICU (Clermont-Ferrand)	12 (18.5)
Neurosurgical ICU (Clermont-Ferrand)	10 (15.4)
Surgical ICU (Clermont-Ferrand)	11 (16.9)
Medical-surgical ICU (Vichy)	3 (4.6)
Medical-surgical ICU (Montluçon)	4 (6.2)
Professional status	
Junior	34 (52.3)
Senior	31 (47.7)
Age (years)	33.6 ± 9.6
Number of years of ICU experience	2 [1; 8]
[1, 2]	33 (50.8)
]2–5]	13 (20.0)
>5	19 (29.2)
Male gender	47 (72.3)
Intensivists with training in cost assessment	2/64 (3.1)
Ability to estimate the cost of their prescriptions (/100) *	30 [15; 50]
<40	41 (63.1)
[40–60]	17 (26.1)
>60	7 (10.8)
Feeling aware of the financial impact of their prescriptions (/100) *	50 [27; 71]
<40	24 (36.9)
[40–60]	19 (29.2)
>60	22 (33.9)
Feeling aware of the environmental impact of their prescriptions (/100) *	50 [30; 65]
<40	21 (32.3)
[40–60]	26 (40.0)
>60	18 (27.7)
Number of patients assessed per intensivist	
3	34 (52.3)
4	23 (35.4)
5	8 (12.3)

Data are presented as the number of patients (column percentages), as mean ± standard deviation, or as median [25th; 75th percentiles]. ICU: intensive care unit. * Self-assessment using visual analogue scales ranging from 0 (entirely incapable/insensitive) to 100 (very capable/sensitive) with < 40/100 corresponding to low ability or awareness, 40–60/100 corresponding to intermediate ability or awareness, and > 60/100 corresponding to high ability or awareness

**Table 2:** Characteristics of the patients

	(*n* = 133)
Intensive care units	
Cancer centre (Clermont-Ferrand)	19 (14.3)
Neurosurgical ICU (Reunion Island)	22 (16.5)
Medical-surgical ICU (Clermont-Ferrand)	26 (19.6)
Neurosurgical ICU (Clermont-Ferrand)	24 (18.0)
Surgical ICU (Clermont-Ferrand)	27 (20.3)
Medical-surgical ICU (Vichy)	6 (4.5)
Medical-surgical ICU (Montluçon)	9 (6.8)
Number of prescriptions per patient	
Overall prescriptions	50 [35; 72]
Drugs	15 [10; 20]
Medical devices	25 [15; 39]
Laboratory tests	10 [7; 12]
Imaging modalities	1 [0; 1]
Waste weight (kg)	
Total	2.4 [1.6; 3.8]
Household-like waste	1.7 [1.0; 2.7]
Waste with infectious risks	0.3 [0.0; 1.4]
Cost per patient (€)	
Overall cost	174 [120; 237]
Drugs	20 [8; 43]
Medical devices	10 [3; 21]
Laboratory tests	104 [77; 137]
Imaging modalities	21 [0; 21]
Waste	1.0 [0.6; 2.2]

Data are presented as the number of patients (column percentages), or as median [25th; 75th percentiles]. ICU: intensive care unit

The estimated overall cost was significantly higher than the real overall cost: €330 [170; 620] vs. €178 [124; 239], respectively, ES: 0.67, 95% CI: 0.49 to 0.86, *p* < 0.001. The distribution of estimated costs according to the real costs and the intensivist’s status is shown in Additional file 2: Fig.S1. Seventy estimations (29.9%, 95% CI: 24.1 to 36.2%) were accurate. The estimated cost was significantly higher than the real cost for the categories of drugs, medical devices, imaging modalities and waste, and lower than the real cost for laboratory tests (Table 3).

**Table 3:** Difference between real and estimated costs

	Real costs (€)	Estimated costs (€)	ES [95%CI]	p
Total (*n* = 234)	178 [124; 239]	330 [170; 620]	0.67 [0.49; 0.86]	< 0.001
Drugs (*n* = 234)	21 [8; 45]	93 [40; 230]	0.57 [0.39; 0.76]	< 0.001
Medical devices (*n* = 231)	10 [3; 23]	50 [20; 100]	0.64 [0.45; 0.83]	< 0.001
Laboratory tests (*n* = 228)	106 [79; 138]	65 [30; 120]	-0.23 [-0.41; -0.05]	< 0.001
Imaging modalities (*n* = 119)	21 [21; 25]	30 [20; 70]	0.59 [0.33; 0.85]	< 0.001
Waste (*n* = 230)	1.1 [0.6; 2.3]	40 [15; 100]	0.81 [0.62; 0.99]	< 0.001

Data are presented as median [25th; 75th percentiles]. CI: confidence interval, ES: effect size. The sample sizes vary depending on the category, because not all patients were concerned

The analyses of the factors associated with an accurate estimation of the overall cost and the difference in overall cost (estimated overall cost minus real overall cost) are shown in Tables 4 and 5, respectively. Being aware of the financial impact of the prescriptions (> 60/100) was associated with a better estimate of the overall cost compared to not being particularly aware (40–60) (OR: 5.05, 95CI: 1.47 to 17.4, *p* = 0.01) (Table 4). Self-predicted ability to accurately estimate the cost of prescriptions (ability to estimate > 60/100) was associated with a poorer estimate compared to not feeling particularly able (40–60) (OR: 0.11, 95%CI: 0.02 to 0.71, *p* = 0.02) (Table 4). Senior status, male sex and being aware of the financial impact of the prescriptions were associated with a lower overall cost difference (Table 5).

**Table 4:** Factors associated with accurate estimate (between 50 and 150% of the real cost) of the overall cost

	Inaccurate estimate (*n* = 164)	Accurate estimate (*n* = 70)	OR [95%CI]	p
Professional status				
Junior	82/122 (67.2)	40/122 (32.8)	Ref.	
Senior	82/112 (73.2)	30/112 (26.8)	0.73 [0.26; 2.07]	0.55
Age (years)	34.0 ± 10.0	33.8 ± 10.0	0.99 [0.94; 1.05]	0.88
Number of years of ICU experience	3 [1; 8]	3 [1; 10]	1.01 [0.94; 1.06]	0.96
[1, 2]	81/115 (70.4)	34/115 (29.6)	Ref.	
]2–5]	34/48 (70.8)	14/48 (29.2)	1.01 [0.23; 4.37]	0.99
>5	49/71 (69.0)	22/71 (31.0)	1.16 [0.35; 3.89]	0.81
Gender				
Male	113/170 (66.5)	57/170 (33.5)	Ref.	
Female	51/64 (79.7)	13/64 (20.3)	0.38 [0.11; 1.30]	0.12
Ability to estimate the cost of their prescriptions (/100) *	25 [12; 50]	40 [20; 50]	1.02 [0.99; 1.04]	0.11
[40–60]	32/66 (48.5)	34/66 (51.5)	Ref.	
<40	112/144 (77.8)	32/144 (22.2)	0.19 [0.06; 0.54]	0.002
>60	20/24 (83.3)	4/24 (16.7)	0.11 [0.02; 0.71]	0.02
Feeling aware of the financial impact of their prescriptions (/100) *	50 [25; 60]	70 [50; 75]	1.03 [1.01; 1.05]	0.006
[40–60]	56/71 (78.9)	15/71 (21.1)	Ref.	
<40	68/85 (80.0)	17/85 (20.0)	0.89 [0.26; 3.09]	0.86
>60	40/78 (51.3)	38/78 (48.7)	5.05 [1.47; 17.4]	0.01
Feeling aware of the environmental impact of their prescriptions (/100) *	50 [30; 65]	50 [30; 71]	1.01 [0.99; 1.03]	0.51
[40–60]	69/95 (72.6)	26/95 (27.4)	Ref.	
<40	53/75 (70.7)	22/75 (29.3)	0.96 [0.26; 3.50]	0.95
>60	42/64 (65.6)	22/64 (34.4)	1.30 [0.34; 4.97]	0.70

Data are presented as the number of patients (row percentages), as mean ± standard deviation, or as median [25th; 75th percentiles]. CI: confidence interval, ICU: intensive care unit, OR: odds ratio, Ref: reference. * Self-assessment using visual analogue scales ranging from 0 (entirely incapable/insensitive) to 100 (very capable/sensitive) with < 40/100 corresponding to low ability or awareness, 40–60/100 corresponding to intermediate ability or awareness, and > 60/100 corresponding to high ability or awareness

**Table 5:** Factors associated with the difference in overall cost (estimated cost minus real cost)

	Difference in overall cost (€)	p
Professional status		
Junior (*n* = 122)	183 [15; 507]	0.04
Senior (*n* = 112)	109 [-17; 348]	
Age (*n* = 234)	-0.14	0.24
Number of years of ICU experience (*n* = 234)	-0.12	0.31
[1, 2] (*n* = 115)	184 [20; 507]	0.06
]2–5] (*n* = 48)	154 [0; 507]	
>5 (*n* = 71)	96 [-18; 290]	
Gender		
Male (*n* = 170)	122 [-6; 351]	0.008
Female (*n* = 64)	250 [50; 624]	
Intensivists with training in cost assessment		
No (*n* = 223)	137 [2; 453]	0.35
Yes (*n* = 8)	229 [140; 265]	
Ability to estimate the cost of their prescriptions * (*n* = 234)	-0.16	0.11
<40 (*n* = 144)	191 [42; 576]	0.10
[40–60] (*n* = 66)	28 [-25; 211]	
>60 (*n* = 24)	229 [52; 314]	
Feeling aware of the financial impact of their prescriptions * (*n* = 234)	-0.31	0.006
<40 (*n* = 85)	280 [67; 599]	0.04
[40–60] (*n* = 71)	163 [41; 561]	
>60 (*n* = 78)	35 [-19; 225]	
Feeling aware of the environmental impact of their prescriptions * (*n* = 234)	0.06	0.78
<40 (*n* = 75)	105 [-18; 475]	0.31
[40–60] (*n* = 95)	166 [14; 312]	
>60 (*n* = 64)	194 [0; 705]	

Data are presented as median [25th; 75th percentiles], or as Spearman’s rank correlation coefficient. ICU: intensive care unit. In the first column, the number of estimates are indicated in parentheses. * Self-assessment using visual analogue scales ranging from 0 (entirely incapable/insensitive) to 100 (very capable/sensitive) with < 40/100 corresponding to low ability or awareness, 40–60/100 corresponding to intermediate ability or awareness, and > 60/100 corresponding to high ability or awareness

The factors associated with a good estimate according to each category are shown in Additional file 2: Tables S1, S2.S3,S4.and S5. Only estimates for patients who received prescriptions for each category were considered.

## Discussion

Our study clearly indicates that both junior and senior intensivists have a poor cost-awareness in their daily clinical practice. They overestimated the overall cost and the cost of drugs, imaging modalities, medical device and waste. In contrast, they underestimated the cost of laboratory tests. Being aware of the financial impact of prescriptions was associated with an accurate estimate, while feeling able to make a good cost estimation was inversely associated with an accurate estimate.

Previous studies reported that the price of the cheapest devices/products was overestimated, while that of the most expensive ones was underestimated [7, 8, 15]. The overestimation of costs results in budgetary imbalance if the examination or the medication is not justified [15]. In our study, the median price of €106 proposed by the intensivists for laboratory tests, which was the most expensive category, was an underestimation. In contrast, other, cheaper categories were overestimated. Our findings are consistent with those of the literature. However, the overall cost of an intensive care day was widely overestimated. This unexpected result can be explained by differences in methodology. Previous studies assessed physicians’ cost estimates of a list of drugs, procedures, imaging modalities, including sometimes the price of magnetic resonance imaging machines, which are more or less frequently used in the ICU and outside of any clinical context [7, 8, 10]. While some drugs or medical devices are expensive, it seems that their use is ultimately rare, which could explain why their cost was overestimated by physicians. In our study, we assessed the prescription costs of intensivists during a day in their clinical practice. This kind of estimation is more related to real life and helps to identify interesting areas for improving the control of ICU costs in daily practice. Results of cost control strategies such as reducing access to automated prescriptions, eliminating redundancies, and adding the prices of tests or drugs to prescription software are contrasting, with, at best, a modest impact [13, 16–20]. Another effective approach to reduce expenditure on drugs and medical devices is to switch from intravenous to oral administration as soon as possible. Oral drugs are less expensive and avoid the use of plastic for tubing, syringes and trocars. However, everyday medical devices are inexpensive (€10 [3; 23]). From a financial point of view, directing these savings efforts at daily medical devices seems futile and can lead to rationing medical devices, which reduces compliance with good medical practices. A study reported that the cost of community-acquired pneumonia treatment did not appear to be related to clinical severity [21]. Much of the cost resulted from non-compliance with guidelines. These findings raise awareness for promoting cost-effective prescribing practices, particularly following guidelines. Regarding the cost of waste, the intensivists in our study assessed the cost of its management to be more than 30 times the real cost. This major difference could be explained by an assimilation of financial cost with ecological impact. Nowadays, the financial cost of waste is inexpensive but the ecological burden, including the carbon footprint, is probably much higher and, unlike that of a currency, difficult to quantify [14, 22]. In our study, only 28% of intensivists were aware of the environmental impact of their prescription (> 60/100). Awareness of the environmental impact of items used in health care along with the results of studies [23–25] could contribute to changing practices to reduce the adverse environmental consequences. Awareness seems to be the best way to improve practices. In our entire sample, only two intensivists had received training in cost assessment, a rate similar to that in a French study published in 2015 [7]. Training for physicians in cost management is underdeveloped probably because it has a limited impact [16, 26–28]. Our study shows that being aware of the financial impact of prescriptions, in contrast to feeling able to make an accurate estimate, was a factor associated with a good estimation. However, a study reported that to expand clinicians ‘cost consciousness had limited impact on clinical decision-making [29]. This suggests that several strategies will need to be deployed to have a substantial impact on cost control. Awareness of different human impacts on resources or climate is the main lever used by several countries to encourage citizens including physicians to take greater responsibility in changing their practices [11, 24]. Sobriety is now required in all human activities including medicine. We could call this momentum the “Auvergnat syndrome”. Auvergne is a French region whose inhabitants had the reputation of being cautious with their money. In the future, however, frugality is probably a quality that could save our environment.

Our study has several limitations. First, we cannot rule out that the data collection was not exhaustive. Some medical devices used during emergency care may have been omitted. Secondly, the data on waste were underestimated because only the bins in patients’ room were weighed. The shared bins in the ICU, which are often used to dispose of drug containers or medical devices, were not weighed. This information was made clear to participants. Thirdly, the costs for electricity or water were not taken into account because it would have been too difficult with the tools at our disposal to make individual assessments for each patient [30]. The cost of oxygen consumption was not assessed, but is very low (€0.17/m^3^). Fourth, we did not investigate whether all prescriptions were appropriate because any assessment would have been subjective. However, inappropriate prescriptions are patently an unnecessary additional cost. Finally, self-assessments of the ability to estimate the costs of prescriptions, and feeling aware of the financial or environmental impact of prescriptions were declarative and subjective.

## Conclusion

Our prospective observational multicentre study provides evidence that senior and junior intensivists have a poor awareness of costs in their daily clinical practice. They widely overestimated overall and waste costs and underestimated those of laboratory tests. Interestingly, being aware of the financial impact of prescriptions and not feeling able to make an accurate cost estimation was associated with a good final estimate. These findings point to the main areas for improvement that could help achieve the objective of getting the best care at the best cost.

## Electronic supplementary material

Below is the link to the electronic supplementary material.

Supplementary Material 1

Supplementary Material 2
