# Supplementary material for: Cost awareness among intensivists in their daily clinical practice: a prospective multicentre study
Source: Eur J Health Econ. 2024 Mar 13;25(9):1529–37. doi: 10.1007/s10198-024-01686-y (PMC11512858; doi:10.1007/s10198-024-01686-y)
Supplement: Supplementary file 2 — Supplementary Material 2 [file 10198_2024_1686_MOESM2_ESM.docx]

Figure S1: Distribution of estimated costs according to the real costs and the intensivists status.

Table S1: Factors associated with accurate estimate (between 50 and 150% of the real cost) of drug costs.

|  | **Inaccurate**  **estimate**  **(n=194)** | **Accurate**  **estimate**  **(n=40)** | **OR [95CI%]** | **p** |
| --- | --- | --- | --- | --- |
| Professional status |  |  |  |  |
| Junior | 101/122 (82.8) | 21/122 (17.2) | Ref. |  |
| Senior | 93/112 (83.0) | 19/112 (17.0) | 0.99 [0.38; 2.57] | 0.98 |
| Age (years) | 33.8 ± 10.2 | 34.4 ± 9.2 | 1.01 [0.96; 1.06] | 0.79 |
| Number of years of ICU experience |  |  |  |  |
| [1 – 2] | 97/115 (84.3) | 18/115 (15.7) | Ref. |  |
| ]2 – 5] | 38/48 (79.2) | 10/48 (20.8) | 1.36 [0.40; 4.61] | 0.62 |
| >5 | 59/71 (83.1) | 12/71 (16.9) | 1.07 [0.36; 3.21] | 0.90 |
| Gender |  |  |  |  |
| Male | 139/170 (81.8) | 31/170 (18.2) | Ref. |  |
| Female | 55/64 (85.9) | 9/64 (14.1) | 0.73 [0.24; 2.17] | 0.57 |
| Ability to estimate the cost of their prescriptions (/100) * |  |  |  |  |
| [40 – 60] | 54/66 (81.8) | 12/66 (18.2) | Ref. |  |
| <40 | 120/144 (83.3) | 24/144 (16.7) | 0.95 [0.32; 2.80] | 0.92 |
| >60 | 20/24 (83.3) | 4/24 (16.7) | 0.84 [0.15; 4.87] | 0.85 |
| Feeling aware of the financial impact of their prescriptions (/100) * |  |  |  |  |
| [40 – 60] | 61/71 (85.9) | 10/71 (14.1) | Ref. |  |
| <40 | 72/85 (84.7) | 13/85 (15.3) | 1.12 [0.34; 3.68] | 0.86 |
| >60 | 61/78 (78.2) | 17/78 (21.8) | 1.72 [0.53; 5.57] | 0.37 |
| Feeling aware of the environmental impact of their prescriptions (/100) * |  |  |  |  |
| [40 – 60] | 80/95 (84.2) | 15/95 (15.8) | Ref. |  |
| <40 | 60/75 (80.0) | 15/75 (20.0) | 1.34 [0.44; 4.04] | 0.60 |
| >60 | 54/64 (84.4) | 10/64 (15.6) | 0.94 [0.28; 3.12] | 0.92 |
| *Data are presented as the number of patients (row percentages), or as mean ± standard deviation. CI: confidence interval, ICU: intensive care unit, OR: odds ratio, Ref: reference. * Self-assessment using visual analogue scales ranging from 0 (entirely incapable/insensitive) to 100 (very capable/sensitive) with <40/100 corresponding to low ability or awareness, 40-60/100 corresponding to intermediate ability or awareness, and >60/100 corresponding to high ability or awareness.* | | | | |

Table S2: Factors associated with accurate estimate (between 50 and 150% of the real cost) of medical device costs.

|  | **Inaccurate**  **estimate**  **(n=199)** | **Accurate**  **estimate**  **(n=32)** | **OR [95CI%]** | **p** |
| --- | --- | --- | --- | --- |
| Professional status |  |  |  |  |
| Junior | 102/120 (85.0) | 18/120 (15.0) | Ref. |  |
| Senior | 97/111 (87.4) | 14/111 (12.6) | 0.83 [0.27; 2.56] | 0.74 |
| Age (years) | 34.3 ± 10.2 | 30.8 ± 6.4 | 0.95 [0.88; 1.02] | 0.14 |
| Number of years of ICU experience |  |  |  |  |
| [1 – 2] | 96/114 (84.2) | 18/114 (15.6) | Ref. |  |
| ]2 – 5] | 38/47 (80.9) | 9/47 (19.1) | 1.47 [0.38; 5.63] | 0.57 |
| >5 | 65/70 (92.9) | 5/70 (7.1) | 0.48 [0.12; 1.91] | 0.30 |
| Gender |  |  |  |  |
| Male | 149/168 (88.7) | 19/168 (11.3) | Ref. |  |
| Female | 50/63 (79.4) | 13/63 (20.6) | 1.71 [0.46; 6.34] | 0.42 |
| Ability to estimate the cost of their prescriptions (/100) * |  |  |  |  |
| [40 – 60] | 54/66 (81.8) | 12/66 (18.2) | Ref. |  |
| <40 | 123/141 (87.2) | 18/141 (12.8) | 0.33 [0.08; 1.28] | 0.11 |
| >60 | 22/24 (91.7) | 2/24 (8.3) | 0.31 [0.03; 2.71] | 0.29 |
| Feeling aware of the financial impact of their prescriptions (/100) * |  |  |  |  |
| [40 – 60] | 62/70 (88.6) | 8/70 (11.4) | Ref. |  |
| <40 | 72/83 (86.7) | 11/83 (13.3) | 1.43 [0.32; 6.36] | 0.64 |
| >60 | 65/78 (83.3) | 13/78 (16.7) | 2.29 [0.49; 10.6] | 0.29 |
| Feeling aware of the environmental impact of their prescriptions (/100) * |  |  |  |  |
| [40 – 60] | 75/93 (80.6) | 18/93 (19.4) | Ref. |  |
| <40 | 65/74 (87.8) | 9/74 (12.2) | 0.53 [0.14; 2.03] | 0.35 |
| >60 | 59/64 (92.2) | 5/64 (7.8) | 0.45 [0.10; 2.08] | 0.30 |
| *Data are presented as the number of patients (row percentages), or as mean ± standard deviation. CI: confidence interval, ICU: intensive care unit, OR: odds ratio, Ref: reference. * Self-assessment using visual analogue scales ranging from 0 (entirely incapable/insensitive) to 100 (very capable/sensitive) with <40/100 corresponding to low ability or awareness, 40-60/100 corresponding to intermediate ability or awareness, and >60/100 corresponding to high ability or awareness.* | | | | |

Table S3: Factors associated with accurate estimate (between 50 and 150% of the real cost) of laboratory test costs.

|  | **Inaccurate**  **estimate**  **(n=136)** | **Accurate**  **estimate**  **(n=92)** | **OR [95CI%]** | **p** |
| --- | --- | --- | --- | --- |
| Professional status |  |  |  |  |
| Junior | 68/120 (56.7) | 52/120 (43.3) | Ref. |  |
| Senior | 68/108 (63.0) | 40/108 (37.0) | 0.77 [0.34; 1.73] | 0.53 |
| Age (years) | 34.1 ± 9.9 | 33.1 ± 9.6 | 0.99 [0.95; 1.03] | 0.68 |
| Number of years of ICU experience |  |  |  |  |
| [1 – 2] | 67/112 (59.8) | 45/112 (40.2) | Ref. |  |
| ]2 – 5] | 26/47 (55.3) | 21/47 (44.7) | 1.17 [0.40; 3.46] | 0.78 |
| >5 | 43/69 (62.3) | 26/69 (37.7) | 0.91 [0.35; 2.36] | 0.85 |
| Gender |  |  |  |  |
| Male | 103/166 (62.0) | 63/166 (38.0) | Ref. |  |
| Female | 33/62 (53.2) | 29/62 (46.8) | 1.68 [0.67; 4.20] | 0.27 |
| Ability to estimate the cost of their prescriptions (/100) * |  |  |  |  |
| [40 – 60] | 42/65 (64.6) | 23/65 (35.4) | Ref. |  |
| <40 | 80/141 (56.7) | 61/141 (43.2) | 1.63 [0.63; 4.24] | 0.31 |
| >60 | 14/22 (63.6) | 8/22 (36.4) | 1.19 [0.27; 5.38] | 0.82 |
| Feeling aware of the financial impact of their prescriptions (/100) * |  |  |  |  |
| [40 – 60] | 30/69 (43.5) | 39/69 (56.5) | Ref. |  |
| <40 | 51/83 (61.4) | 32/83 (38.6) | 0.42 [0.16; 1.08] | 0.07 |
| >60 | 55/76 (72.4) | 21/76 (27.6) | 0.22 [0.08; 0.61] | 0.004 |
| Feeling aware of the environmental impact of their prescriptions (/100) * |  |  |  |  |
| [40 – 60] | 53/93 (57.0) | 40/93 (43.0) | Ref. |  |
| <40 | 52/74 (70.3) | 22/74 (29.7) | 0.54 [0.20; 1.46] | 0.23 |
| >60 | 31/61 (50.8) | 30/61 (49.2) | 1.51 [0.54; 4.24] | 0.43 |
| *Data are presented as the number of patients (row percentages), or as mean ± standard deviation. CI: confidence interval, ICU: intensive care unit, OR: odds ratio, Ref: reference. * Self-assessment using visual analogue scales ranging from 0 (entirely incapable/insensitive) to 100 (very capable/sensitive) with <40/100 corresponding to low ability or awareness, 40-60/100 corresponding to intermediate ability or awareness, and >60/100 corresponding to high ability or awareness.* | | | | |

Table S4: Factors associated with accurate estimate (between 50 and 150% of the real cost) of imaging modalities costs.

|  | **Inaccurate**  **estimate**  **(n=78)** | **Accurate**  **estimate**  **(n=41)** | **OR [95CI%]** | **p** |
| --- | --- | --- | --- | --- |
| Professional status |  |  |  |  |
| Junior | 42/64 (65.6) | 22/64 (34.4) | Ref. |  |
| Senior | 36/55 (65.5) | 19/55 (34.5) | 0.85 [0.18; 4.05] | 0.84 |
| Age (years) | 32.6 ± 8.7 | 34.0 ± 9.7 | 1.02 [0.93; 1.11] | 0.66 |
| Number of years of ICU experience |  |  |  |  |
| [1 – 2] | 39/58 (67.2) | 19/58 (32.8) | Ref. |  |
| ]2 – 5] | 18/28 (64.3) | 10/28 (35.7) | 1.34 [0.18; 9.74] | 0.77 |
| >5 | 21/33 (63.6) | 12/33 (36.4) | 1.13 [0.17; 7.55] | 0.90 |
| Gender |  |  |  |  |
| Male | 54/84 (64.3) | 30/84 (35.7) | Ref. |  |
| Female | 24/35 (68.6) | 11/35 (31.4) | 0.59 [0.10; 3.51] | 0.56 |
| Ability to estimate the cost of their prescriptions (/100) * |  |  |  |  |
| [40 – 60] | 22/30 (73.3) | 8/30 (26.7) | Ref. |  |
| <40 | 49/77 (63.6) | 28/77 (36.4) | 2.08 [0.31; 13.8] | 0.45 |
| >60 | 7/12 (58.3) | 5/12 (41.7) | 2.84 [0.16; 51.6] | 0.48 |
| Feeling aware of the financial impact of their prescriptions (/100) * |  |  |  |  |
| [40 – 60] | 26/40 (65.0) | 14/40 (35.0) | Ref. |  |
| <40 | 31/45 (68.9) | 14/45 (31.1) | 0.67 [0.10; 4.37] | 0.68 |
| >60 | 21/34 (61.8) | 13/34 (38.2) | 1.11 [0.14; 8.55] | 0.92 |
| Feeling aware of the environmental impact of their prescriptions (/100) * |  |  |  |  |
| [40 – 60] | 34/51 (66.7) | 17/51 (33.3) | Ref. |  |
| <40 | 20/30 (66.7) | 10/30 (33.3) | 0.84 [0.12; 6.12] | 0.87 |
| >60 | 24/38 (63.2) | 14/38 (36.8) | 0.92 [0.13; 6.59] | 0.93 |
| *Data are presented as the number of patients (row percentages), or as mean ± standard deviation. CI: confidence interval, ICU: intensive care unit, OR: odds ratio, Ref: reference. * Self-assessment using visual analogue scales ranging from 0 (entirely incapable/insensitive) to 100 (very capable/sensitive) with <40/100 corresponding to low ability or awareness, 40-60/100 corresponding to intermediate ability or awareness, and >60/100 corresponding to high ability or awareness.* | | | | |

Table S5: Factors associated with accurate estimate (between 50 and 150% of the real cost) of waste costs.

|  | **Inaccurate**  **estimate**  **(n=213)** | **Accurate**  **estimate**  **(n=13)** | **OR [95CI%]** | **p** |
| --- | --- | --- | --- | --- |
| Professional status |  |  |  |  |
| Junior | 114/120 (95.0) | 6/120 (5.0) | Ref. |  |
| Senior | 103/110 (93.6) | 7/110 (6.4) | 1.32 [0.35; 4.97] | 0.68 |
| Age (years) | 33.6 ± 9.8 | 37.0 ± 11.0 | 1.06 [0.98; 1.13] | 0.13 |
| Number of years of ICU experience |  |  |  |  |
| [1 – 2] | 108/113 (95.6) | 5/113 (4.4) | Ref. |  |
| ]2 – 5] | 43/48 (89.6) | 5/48 (10.4) | 4.10 [0.95; 17.8] | 0.06 |
| >5 | 66/69 (95.7) | 3/69 (4.3) | 1.45 [0.30; 7.05] | 0.64 |
| Gender |  |  |  |  |
| Male | 157/166 (94.6) | 9/166 (5.4) | Ref. |  |
| Female | 60/64 (93.7) | 4/64 (6.3) | 0.45 [0.10; 2.08] | 0.31 |
| Ability to estimate the cost of their prescriptions (/100) * |  |  |  |  |
| [40 – 60] | 61/65 (93.8) | 4/65 (6.2) | Ref. |  |
| <40 | 136/142 (95.8) | 6/142 (4.2) | 0.33 [0.07; 1.50] | 0.15 |
| >60 | 20/23 (87.0) | 3/23 (13.0) | 2.94 [0.42; 20.5] | 0.28 |
| Feeling aware of the financial impact of their prescriptions (/100) * |  |  |  |  |
| [40 – 60] | 62/70 (88.6) | 8/70 (11.4) | Ref. |  |
| <40 | 84/84 (100) | 0/84 (0.0) | NE | NE |
| >60 | 71/76 (93.4) | 5/76 (6.6) | NE | NE |
| Feeling aware of the environmental impact of their prescriptions (/100) * |  |  |  |  |
| [40 – 60] | 86/94 (91.5) | 8/94 (8.5) | Ref. |  |
| <40 | 69/73 (94.5) | 4/73 (5.5) | 0.70 [0.16; 3.07] | 0.64 |
| >60 | 62/63 (98.4) | 1/63 (1.6) | 0.42 [0.04; 4.41] | 0.47 |
| *Data are presented as the number of patients (row percentages), or as mean ± standard deviation. CI: confidence interval, ICU: intensive care unit, NE: not estimated, OR: odds ratio, Ref: reference. * Self-assessment using visual analogue scales ranging from 0 (entirely incapable/insensitive) to 100 (very capable/sensitive) with <40/100 corresponding to low ability or awareness, 40-60/100 corresponding to intermediate ability or awareness, and >60/100 corresponding to high ability or awareness.* | | | | |
